# Relationship Between Fluid Administration During the First Three Hours of Sepsis Resuscitation and Mortality: A Multicenter Observational Study

**DOI:** 10.7759/cureus.65480

**Published:** 2024-07-26

**Authors:** Toshikazu Abe, Yutaka Umemura, Hiroshi Ogura, Shigeki Kushimoto, Seitato Fujishima, Atsushi Shiraishi, Daizo Saitoh, Toshihiko Mayumi, Yasuhiro Otomo, Taka-aki Nakada, Satoshi Gando

**Affiliations:** 1 Critical Care, Health Services Research and Development Center, University of Tsukuba, Tsukuba, JPN; 2 Emergency Medicine, Osaka General Medical Center, Osaka, JPN; 3 Trauma and Surgical Critical Care, Osaka General Medical Center, Osaka, JPN; 4 Emergency and Critical Care Medicine, Tohoku University School of Medicine, Sendai, JPN; 5 Critical Care, Center for Preventive Medicine, Keio University School of Medicine, Tokyo, JPN; 6 Emergency Medicine, Emergency and Trauma Center, Kameda Medical Center, Chiba, JPN; 7 Traumatology and Critical Care Medicine, National Defense Medical College, Tokorozawa, JPN; 8 Emergency Medicine, University of Occupational and Environmental Health, Kitakyushu, JPN; 9 Emergency Medicine, Trauma and Acute Critical Care Center, Tokyo Medical and Dental University, Tokyo, JPN; 10 Emergency and Critical Care Medicine, Chiba University, Chiba, JPN; 11 Acute and Critical Care Medicine, Sapporo Higashi Tokushukai Hospital, Sapporo, JPN

**Keywords:** sepsis, fluid resuscitation, sepsis-3 criteria, fluid resuscitation in sepsis, fluid therapy

## Abstract

Background

Timely and effective fluid resuscitation is vital for stabilizing sepsis while avoiding volume overload. We aimed to assess how the administration of a 30 mL/kg bolus fluid affects patients with sepsis within three hours of clinical outcomes.

Methods

This multicenter observational study included adult patients diagnosed with sepsis in 17 intensive care units at tertiary hospitals in Japan between July 2019 and August 2020. The clinical outcomes of patients with sepsis who received ≥30 mL/kg bolus fluid within three hours (30 × 3 group) were compared with those who received <30 mL/kg fluid (non-30 × 3 group).

Results

Of 172 eligible patients, 74 (43.0%) belonged to the 30 × 3 group, and 98 (57.0%) belonged to the non-30 × 3 group. The median Sequential Organ Failure Assessment score was 9 (interquartile range (Q1-Q3): 7-11) in the 30 × 3 group and 7 (Q1-Q3: 4-9) in the non-30 × 3 group (P < 0.01). The 28-day mortality rate was 29.7% in the 30 × 3 group and 12.2% in the non-30 × 3 group (P < 0.01). However, the adjusted odds ratio by the inverse probability of treatment weighting analysis with propensity score for the 28-day mortality rate of the 30 × 3 group compared with that in the non-30 × 3 group was 2.17 (95% confidence interval: 0.85-5.54). Among the propensity score-matched patients, the 28-day mortality rate was 30% in the 30 × 3 (n = 70) and non-30 × 3 (n = 95) groups, respectively (P = 0.72).

Conclusions

Patients with sepsis who received the 30 mL/kg bolus fluid within three hours experienced more severe clinical outcomes. However, it was not associated with the increased odds of the 28-day mortality.

## Introduction

Sepsis is a fatal disease and one of the most common causes of intensive care unit (ICU) admissions [1]. One of the most important keys to its initial resuscitation is fluid management. For patients with sepsis-induced hypoperfusion or septic shock, intravenous crystalloid fluid bolus is necessary in the rescue phase. However, without appropriate optimization, volume overload cannot be avoided, especially in older patients and patients with heart failure in recent years. Thus, timely and effective fluid resuscitation is vital to stabilize sepsis.

The Surviving Sepsis Campaign recommends a 30 mL/kg bolus fluid within three hours of septic shock identification [2]. However, it remains a weak recommendation and has low-quality evidence because the evidence stems from observational studies, which are the source of significant controversy, particularly for patients who are at risk of adverse effects from fluid bolus therapy [3,4]. Nonetheless, this recommendation is still currently regarded as the best practice because of the few available data suggesting that a change is needed.

However, this recommendation should be evaluated using real-world data. Therefore, our study aimed to assess the impact of administering a 30 mL/kg bolus fluid to patients with sepsis within three hours of clinical outcomes in Japan, a country that is highly adherent to sepsis bundles [5,6].

This article was previously presented as a meeting abstract at the ACEP 2022 Annual Scientific Assembly on October 1st, 2022.

## Materials and methods

Design and setting

This research is a multicenter observational study known as the Japanese Association for Acute Medicine (JAAM) MAESTRO (18323) [5]. It was conducted in 17 ICUs in Japan from July 2019 to August 2020.

Participants

The inclusion criteria were the following: (1) aged above 16 years; (2) fulfilled the Sepsis-3 criteria [7]; and (3) diagnosed with only a new-onset infection.

The exclusion criteria were as follows: (1) had a cardiopulmonary arrest on hospital arrival or post-cardiopulmonary arrest resuscitation status; (2) had the limitation of sustained life care during sepsis diagnosis; (3) transferred from other hospitals; and (4) deemed ineligible as study participants by a site investigator.

Data collection and definitions

Data were extracted from the MAESTRO database and compiled by the MAESTRO investigators. It was convenience sampling. Data such as demographics, comorbidities, vital signs, laboratory data, and infection site were collected as part of the clinical routine workup. We also obtained data on adherence to sepsis care bundles (specifically, the hour-1 bundle). The primary outcome was mortality within 28 days, and the secondary outcomes were ventilator-free days and ICU-free days, length of hospital stay (LOS), and place after discharge.

Sepsis was recognized according to the clinical judgment of the physician-in-charge during the initial evaluation. The physician-in-charge also recorded the timestamp in the database.

Analysis

The clinical characteristics and outcomes of patients with sepsis who received a 30 mL/kg bolus fluid within three hours of sepsis recognition were compared with those who did not. Subsequently, the impact of administering a 30 mL/kg bolus fluid within three hours on a risk-adjusted 28-day mortality rate was evaluated by logistic regression analysis, adjusted by inverse probability of treatment weighting (IPTW) analysis with propensity score. In the IPTW analysis, the stabilized IPTW weight was calculated using the predicted probabilities from the propensity score model. Propensity score matching (average treatment effects) was then used to compare them. In the propensity score matching analysis, a matched control sample was created using the nearest-neighbor method with replacement. In all analyses, the adjusted variables, which were specified a priori based on clinical experience and prior studies, were the patient’s age, sex, admission source (emergency department or ICU), the Charlson comorbidity index, mechanical ventilation use, and each organ score within the Sequential Organ Failure Assessment (SOFA) score. Two-tailed P-values of less than 0.05 were considered statistically significant. All statistical data were analyzed using the STATA software version 16.1 (Stata Corp., College Station, TX, USA).

## Results

Out of 172 eligible patients, 74 (43.0%) received 30 mL/kg bolus fluid within three hours (30 × 3 group), and 98 (57.0%) received less than 30 mL/kg bolus fluid within the same period (non-30 × 3 group). Patients with obesity (body mass index (BMI) ≥ 25 kg/m^2^) were less likely to receive 30 mL/kg bolus fluid than those with normal weight (P = 0.04) (Table 1).

**Table 1:** Demographics and characteristics comparing septic patients with and without the 30 by 3, before and after propensity-score matching Reported counts (proportions) for categorical variables and median (interquartile range) for continuous variables. Missing data: Urine output in 24 h=4 SMD: standardized mean difference; BMI: body mass index; COPD: chronic obstructive pulmonary disease; AIDS: acquired immune deficiency syndrome; SOFA: Sequential (sepsis-related) Organ Failure Assessment; NPPV: noninvasive positive pressure ventilation; NA: not applicable ^†^ The adjusted variables of the propensity score model were the patient’s age, sex, admission source (emergency department or ICU), the Charlson comorbidity index, mechanical ventilation use, and each organ score within the SOFA score

Variables		Before adjusted			After adjusted		
		30 by 3 group, n (%)	non-30 by 3 group, n (%)	SMD	30 by 3 group, n (%)	non-30 by 3 group, n (%)	SMD
		74 (43.0%)	98 (57.0%)		70 (42.4%)	95 (57.6%)	
Age, yr^†^		73.11 ± 15.08	72.55 ± 13.15	1.11	70.81 ± 17.63	72.4 ± 12.92	0.10
Sex^†^	Male	45 (60%)	70 (72.2%)	0.26	50 (66.1%)	65 (67%)	0.02
Admission source^†^	From emergency department	74 (98.7%)	94 (96.9%)	0.12	74 (98.9%)	95 (97.7%)	0.09
BMI, kg/m^2^		20.55 ± 4.28	22.46 ± 4.76	1.00	21.17 ± 4.58	22.69 ± 4.69	0.33
Obesity (BMI>=25)		11 (14.9%)	27 (28.1%)	0.33	13 (17.8%)	29 (30.1%)	0.29
Clinical frailty scale		3.99 ± 1.75	3.74 ± 1.59	0.78	3.82 ±. 1.57	4.04 ± 1.75	0.13
Charlson comorbidity index^†^	0	19 (25.3%)	28 (28.9%)	0.08	14 (18.2%)	28 (29%)	0.26
	1-2	37 (49.3%)	39 (40.2%)	0.18	43 (56.9%)	35 (36%)	0.43
	3-4	12 (16%)	17 (17.5%)	0.04	10 (12.8%)	22 (22.4%)	0.25
	4<	7 (9.3%)	13 (13.4%)	0.13	9 (12.1%)	12 (12.6%)	0.02
Coexisting conditions	Myocardial infarction	5 (6.7%)	8 (8.2%)	0.06	4 (5.8%)	11 (11%)	0.19
	Congestive heart failure	6 (8%)	12 (12.4%)	0.14	6 (8.3%)	12 (12.3%)	0.13
	Peripheral vascular disease	0 (0%)	5 (5.2%)	0.33	0 (0%)	6 (6.5%)	0.37
	Cerebrovascular disease	9 (12%)	7 (7.2%)	0.16	10 (12.9%)	6 (6.4%)	0.22
	Dementia	9 (12%)	10 (10.3%)	0.05	9 (12.5%)	10 (10.4%)	0.06
	COPD	6 (8%)	6 (6.2%)	0.07	3 (4.3%)	10 (10%)	0.22
	Connective tissue disease	5 (6.7%)	4 (4.1%)	0.11	10 (13.1%)	6 (6.3%)	0.23
	Peptic ulcer disease	3 (4%)	8 (8.2%)	0.18	3 (3.4%)	12 (11.9%)	0.32
	Mild liver disease	3 (4%)	8 (8.2%)	0.18	4 (4.8%)	10 (10.5%)	0.21
	Diabetes mellitus without organ damage	22 (29.3%)	22 (22.7%)	0.15	23 (30.5%)	21 (21.6%)	0.21
	Diabetes mellitus with organ damage	3 (4%)	8 (8.2%)	0.18	5 (6%)	9 (9%)	0.11
	Chronic kidney disease	9 (12%)	16 (16.5%)	0.13	9 (12.2%)	18 (18.3%)	0.17
	Hemiplegia	1 (1.3%)	1 (1%)	0.03	1 (0.7%)	1 (0.7%)	0.00
	Malignancy (solid)	8 (10.7%)	10 (10.3%)	0.01	7 (9.6%)	9 (9%)	0.02
	Malignancy (leukemia)	0 (0%)	2 (2.1%)	0.21	0 (0%)	2 (2.1%)	0.21
	Malignancy (malignant lymphoma)	2 (2.7%)	1 (1%)	0.12	7 (8.7%)	1 (0.6%)	0.39
	Metastatic tumor	4 (5.3%)	6 (6.2%)	0.04	4 (5.7%)	4 (4.4%)	0.06
	Moderate to severe liver disease	0 (0%)	2 (2.1%)	0.21	0 (0%)	2 (2%)	0.20
	AIDS	0 (0%)	0 (0%)	NA	0 (0%)	0 (0%)	NA
Suspected site of infection	Lung	29 (38.7%)	48 (49.5%)	0.22	31 (40.9%)	45 (46.2%)	0.11
	Abdomen	16 (21.3%)	17 (17.5%)	0.10	19 (24.8%)	21 (22.2%)	0.06
	Urinary tract	19 (25.3%)	11 (11.3%)	0.37	15 (20.4%)	11 (11.8%)	0.24
	Skin/Soft tissue	4 (5.3%)	7 (7.2%)	0.08	2 (2%)	5 (4.7%)	0.15
	Implant device	0 (0%)	1 (1%)	0.14	0 (0%)	1 (1%)	0.14
	Central nervous system	0 (0%)	2 (2.1%)	0.21	0 (0%)	1 (1.5%)	0.18
	Endocarditis	0 (0%)	1 (1%)	0.14	0 (0%)	2 (1.9%)	0.20
	Intravenous catheter	0 (0%)	1 (1%)	0.14	0 (0%)	1 (1%)	0.14
	Other	2 (2.7%)	2 (2.1%)	0.04	2 (3.2%)	3 (3.1%)	0.00
	Unidentified	5 (6.7%)	7 (7.2%)	0.02	7 (8.8%)	7 (6.7%)	0.08
Positive blood cultures		42 (56%)	51 (52.6%)	0.07	40 (53%)	50 (51.2%)	0.04
Shock		57 (76%)	37 (38.1%)	0.83	54 (72.1%)	45 (46.1%)	0.55
Baseline SOFA score		0.37 ± 1.94	0.62 ± 1.4	1.46	0.26 ± 1.58	0.84 ± 1.63	0.36
SOFA score^†^		9.43 ± 2.78	7.34 ± 2.94	1.19	8.38 ± 2.79	8.13 ± 3.09	0.08
NPPV use		1 (1.3%)	2 (2.1%)	0.06	1 (1%)	2 (2.1%)	0.09
Mechanical ventilation use^†^		35 (46.7%)	29 (29.9%)	0.35	27 (36.4%)	33 (34%)	0.05
Total amount of fluid in 6 h (mL)		5322 ± 4447	1520 ± 896	0.70	4698 ± 3831	1642 ± 932	1.10
Urine output in 24 h (mL)		1254 ± 1000	1171 ± 853	0.25	1509 ± 1148	1150 ± 877	0.35

The 30 × 3 group had more shock cases (75.7%) than the non-30 × 3 group (38.8%) (P < 0.01). The median SOFA score in the 30 × 3 group was 9 (interquartile range (Q1-Q3): 7-11), whereas that in the non-30 × 3 group was 7 (Q1-Q3: 4-9). Among the patients who showed vasopressor indications (n = 92), 40/53 (75.5%) received vasopressor initiation within one hour in the 30 × 3 group and 22/39 (56.4%) in the non-30 × 3 group (P = 0.05). Moreover, mechanical ventilation including noninvasive positive pressure ventilation (NPPV) was needed more in the 30 × 3 group than in the non-30 × 3 group (P = 0.05). The median total fluid amount after six hours was 3675 (Q1-Q3: 2740-5700 mL) for patients in the 30 × 3 group and 1380 (Q1-Q3: 720-2100 mL) for those in the non-30 × 3 group (P < 0.01).

The ICU-free days were shorter in the 30 × 3 group than in the non-30 × 3 group (median (interquartile range): 6.5 (0-18) days vs. 19.5 (6-24) days, P < 0.01). (Table 2).

**Table 2:** Outcomes comparing septic patients with and without the 30 by 3 Reported counts (proportions) for categorical variables and median (interquartile range) for continuous variables. ICU: intensive care unit; NA: not applicable; PS: propensity score

Outcomes		30 by 3 group	non-30 by 3 group	P-value
n (%)		74 (43.0)	98 (57.0)	
Ventilator free days		16 (0-24)	23.5 (5-28)	<0.01
ICU free days		6.5 (0-18)	19.5 (6-24)	<0.01
Length of hospital stay		18 (9-37)	21 (11-49)	0.17
Crude 28-day mortality (n=172)		22 (29.7)	12 (12.2)	<0.01
Crude In-hospital mortality (n=172)		27 (36.5)	14 (14.3)	<0.01
Place after discharge (n=131)	transfer	28 (59.6)	45 (53.6)	0.51
	home	19 (40.4)	39 (46.4)	
PS matching cohort	n (%)	70 (42.4)	95 (57.6)	NA
28-day mortality	%	30	30	0.72

**Figure 1:**
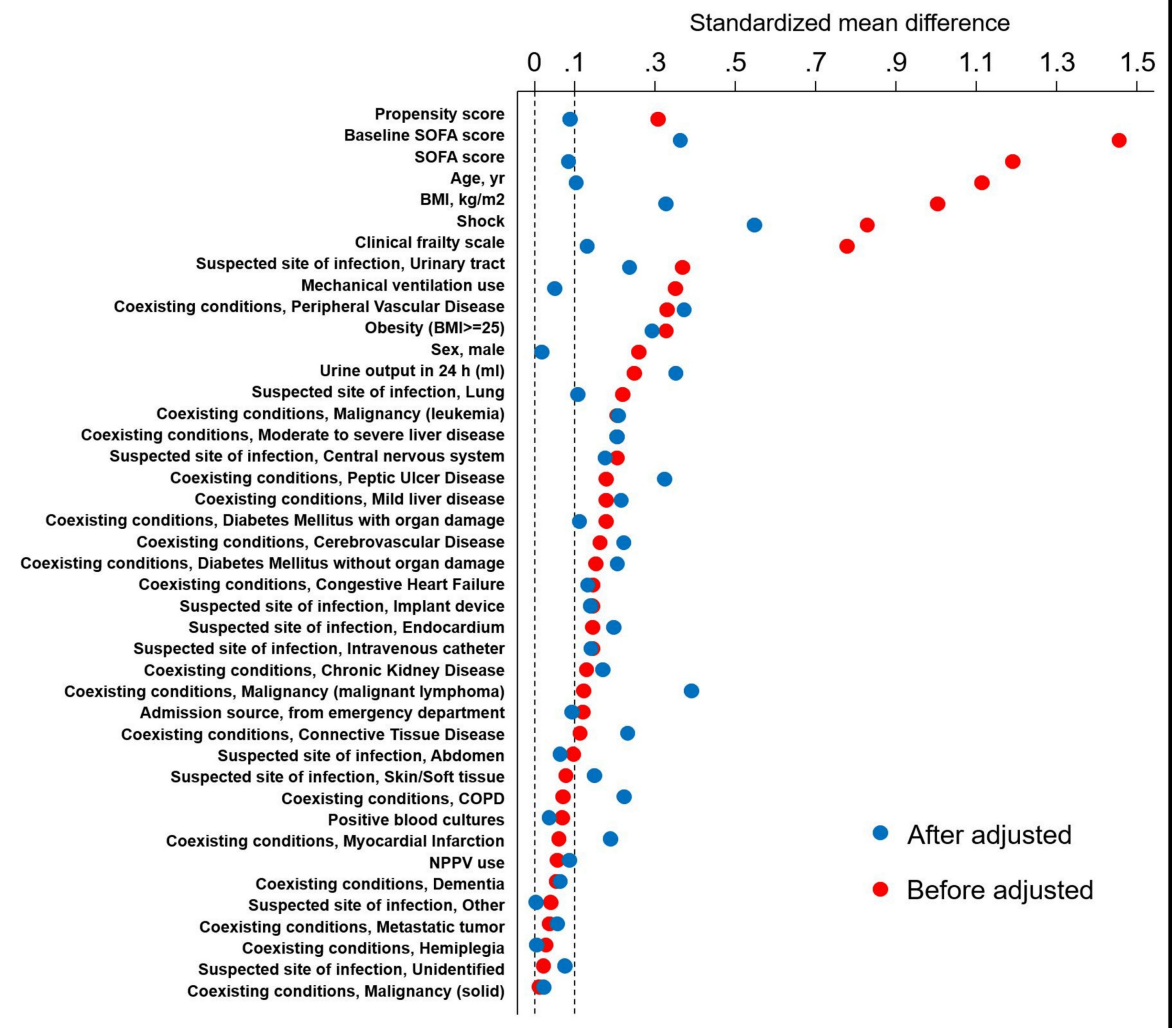
Love plot showing balance covariate before and after propensity score matching SOFA: Sequential Organ Failure Assessment; BMI: body mass index; COPD: chronic obstructive pulmonary disease; NPPV: noninvasive positive pressure ventilation

However, LOS was not significantly different between the two groups (P = 0.17). The crude 28-day mortality rate in the 30 × 3 group was 29.7%, whereas that in the non-30 × 3 group was 12.2% (P < 0.01). Furthermore, the crude in-hospital mortality rate was 36.5% in the 30 × 3 group and 14.3% in the non-30 × 3 group (P < 0.01) and the odds ratio for the 28-day mortality rate of the 30 × 3 group in comparison with that of the non-30 × 3 group was 3.63 (95% confidence interval: 1.72-7.70). However, the adjusted odds ratio for the 28-day mortality rate of the 30 × 3 group in comparison with that of the non-30 × 3 group was 2.17 (95% confidence interval: 0.85-5.54). Among the propensity score-matched patients, the 28-day mortality rate was 30% in the 30 × 3 (n = 70) and non-30 × 3 (n = 95) groups, respectively (P = 0.72). Figure 1 shows the balancing covariates before and after propensity score matching.

## Discussion

In our retrospective observational study, most patients were older adults with multiple comorbidities but with very mild frailty. Our study showed that 30 mL/kg bolus fluid within three hours of clinical outcomes was not associated with the increased risk of mortality even in patients with sepsis admitted to the ICU. Therefore, 30 mL/kg bolus fluid within three hours is an important resuscitation in the rescue phase of sepsis.

Patients with a history of clinically likely fluid retention resulting from a disease, such as heart failure or renal failure, tend to refrain from aggressive fluid resuscitation such as the 30 mL/kg bolus fluid within three hours [4,8]. In our results, patients with this history were not less likely to achieve such aggressive treatment. Conversely, patients with obesity were less likely to achieve it, consistent with the results of a previous study [4]. Perhaps, patients who appeared overweight or had fluid retention tended to refrain from aggressive fluid, or some physicians decided fluid volume by ideal body weight rather than the actual body weight. The difference in the study population in each study might have been influenced by the physician’s decision. However, in our study, aggressive treatment could have been used to provide further fluid resuscitation regardless of the patient’s background by reference to the results of previous studies [4,8]. Japan has been proven to have a very high adherence to guidelines and bundles in sepsis [5,6]. As shown in previous studies, the administration of 30 mL/kg bolus fluid within three hours did not increase in-hospital mortality for patients at risk of adverse effects from fluid bolus therapy in our study.

Although our study did not show a decrease in in-hospital mortality, the 30 × 3 group was clearly more severe and had a higher in-hospital mortality rate. After adjusting for patient severity, in-hospital mortality did not significantly differ between the groups. Thus, this fluid bolus therapy apparently tended to improve the outcome. This study is consistent with many recent studies demonstrating the mortality benefit of early fluid administration in septic shock [8-11], although another study did not find any benefit [12]. However, our study did not find a decrement in LOS, contrary to some previous studies [8,10]. The relationship between fluid and LOS is still controversial because LOS may be more influenced by social factors. Otherwise, the effects of fluid retention may be more pronounced during a longer clinical course. The optimal target volume not only for the rescue phase but also for a longer clinical course remains unknown and will require further studies.

All the above-mentioned previous studies discussed that fluid timing matters [4,8-11]. In other previous studies, vasopressor was administered earlier to reduce fluid volume, but no survival benefit was observed [13,14]. In our study, the total volume of fluid in six hours at 30 mL/kg bolus fluid within three hours was not so high, suggesting that the fluid was managed using dynamic parameters [15,16]. The adverse effects of fluid overload may occur after the rescue phase in patients with poor response to fluids. Fluid resuscitation in the rescue phase is distinctly different from fluid therapy provided in the optimization and stabilization phase [17]. Tissue ischemia caused by shock is associated with high mortality despite brief periods of arterial hypotension in the rescue phase of sepsis. Early fluid bolus therapy for patients with shock may decrease the consequences of the need for subsequent fluid [10]. This point is even true in patients who have a history of congestive heart failure or chronic renal failure. Our results validated previous studies that early fluid resuscitation improves survival in the rescue phase.

This study has several limitations. First, this study has a retrospective design, which could not completely identify the causal inference. Although more severe patients could have received more fluid, no evidence suggests that they had poor outcomes resulting from fluid volume. Residual confounders may have existed. Second, this study was conducted only in Japan. Japan has a very high rate of sepsis bundle adherence. Considering that the influence of other components of the sepsis bundle also plays a role in the patient’s prognosis, the impact of the early aggressive fluid administration may not be recognizable if adherence to other components of the bundle is low [5,6]. Third, the data in this study were from 2019 onwards, when the sepsis bundles were already well known. Possibly, some effectiveness could have already been achieved with early aggressive fluid administration regardless of the patient’s disease history. Therefore, this study is a validation study. Fourth, it involved a small sample size. This is due to not only convenience sampling but also the coronavirus pandemic. Fifth, the mean SOFA score between groups differed only by two although there were substantially more patients in shock on vasopressors in the group that received 30 mL/kg. It might have underestimated the severity of the group. Finally, only half of our patients had shock, as in a previous study [4]; it may have included patients without the indications of the bolus fluid therapy of 30 mL/kg within three hours. However, this population could have shown the real-world nature because hypoperfusion for patients with sepsis is difficult to recognize.

## Conclusions

Patients with sepsis who received the 30 mL/kg bolus fluid within three hours experienced more severe clinical outcomes. However, this aggressive therapy was not associated with the increased odds of the 28-day mortality rate. Therefore, suggestively, timely, and adequate fluid resuscitation can improve patients’ survival.
